# Age-Specific Risk of Herpes Zoster in Adults Aged ≥18 Years With Comorbid Conditions—A Retrospective Cohort Study in the United States

**DOI:** 10.1093/cid/ciag095

**Published:** 2026-03-27

**Authors:** Rachel A Cohen, Driss Oraichi, Agnes Mwakingwe-Omari, Bruno Anspach, Desmond Curran, Huifeng Yun

**Affiliations:** GSK, Rockville, Maryland, USA; GSK, Rockville, Maryland, USA; GSK, Rockville, Maryland, USA; GSK, Rockville, Maryland, USA; GSK, Wavre, Belgium; GSK, Rockville, Maryland, USA

**Keywords:** herpes zoster, comorbidity, adult, incidence, United States

## Abstract

**Background:**

Older (≥50 years of age [YoA]) and immunocompromised adults are at higher risk of herpes zoster (HZ). Those with comorbidities are also at increased risk, however, recent US evidence is lacking, especially among 18–49 YoA.

**Methods:**

This US retrospective cohort study (2015–2022, Merative MarketScan Commercial and Medicare Supplemental) compared HZ incidence rates (IRs) in younger immunocompetent adults (18–49 YoA) with comorbidities (asthma, chronic kidney disease [CKD], chronic obstructive pulmonary disease [COPD], depression, diabetes, stress, and trauma) versus immunocompetent adults 50–59 YoA without comorbidities. Adjusted HZ IR ratios (aIRRs) versus 50–59 YoA adults were compared using a predetermined 0.62 non-inferiority margin for the aIRR 95% confidence interval lower limit (95% CI LL). aIRRs were classified as comparable (aIRR 95% CI LL > 0.62 and ≤1.0), significantly higher (aIRR 95% CI LL > 1.0), or inconclusive (any other result). HZ case: diagnostic code B02.2x plus oral antiviral ±7 days. Sensitivity analyses investigated HZ IRs by number of comorbid conditions (1, 2, or ≥3).

**Results:**

HZ IRs were significantly higher (aIRR 95% CI LL > 1.0) from 30 to 39 YoA (aIRR; 95% CI) in asthma (1.19; 1.10–1.29), COPD (1.31; 1.22–1.40), depression (1.31; 1.22–1.40), diabetes (1.18; 1.06–1.32), stress (1.28; 1.11–1.47), and trauma (1.25; 1.17–1.34) populations than immunocompetent 50–59 YoA and from 50 to 59 YoA in CKD (1.50; 1.28–1.77). In sensitivity analyses, HZ IR appeared to increase with more comorbid conditions and age.

**Conclusions:**

The study found that younger adults (30+ YoA) with certain comorbidities have a higher HZ risk than immunocompetent 50–59 YoA adults.

## Teaser

A retrospective US study in immunocompetent adults found that adults aged ≥30 years with asthma, chronic obstructive pulmonary disease, depression, diabetes, stress, and trauma had increased herpes zoster incidence rates versus those without these comorbid conditions, thus may benefit from vaccination.

## Graphical Abstract

**Graphical Abstract:**
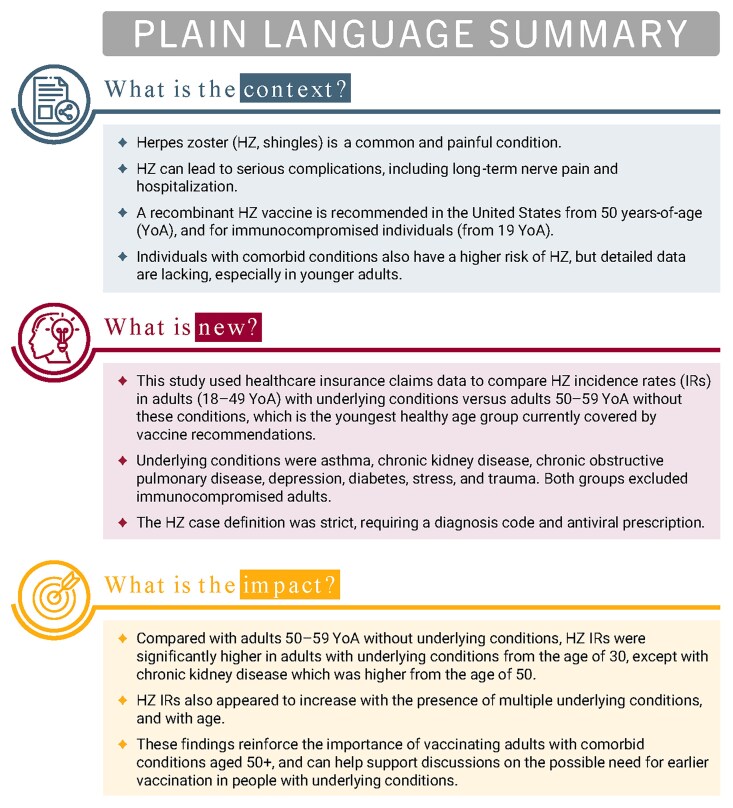

Varicella-zoster virus (VZV) causes varicella (chickenpox) during primary infection and can later reactivate as herpes zoster (HZ, shingles) when VZV-specific cell-mediated immunity declines [1]. Herpes zoster appears as a painful, pruritic rash resolving in 2–4 weeks. Postherpetic neuralgia (PHN), chronic neuropathic pain lasting ≥90 days, is the most common HZ complication, occurring in 10%–18% of adults with HZ [2] and up to 30% of adults ≥80 years of age (YoA) with HZ [3].

Around 1 million new HZ cases occur annually in the United States (US), with incidence rising with age [1]. Adults who are immunocompromised or have certain underlying conditions, for which the immunomodulating effect is less well understood, are at increased risk of HZ [1, 4]. An adjuvanted recombinant HZ vaccine (RZV) is currently recommended in the US for adults ≥50 YoA [5, 6] and for adults ≥19 YoA at increased risk of HZ due to immunodeficiency or immunosuppression caused by a known disease or therapy [7, 8].

Evidence shows that some chronic or comorbid conditions may also increase the risk of HZ, severe HZ, or recurrent HZ [4, 9, 10]. A retrospective cohort study in Germany reported around a 30% higher HZ risk in adults with asthma or chronic obstructive pulmonary disease (COPD) compared with adults with no underlying conditions, while younger adults (18–49 YoA) with a chronic condition had the highest risk of recurrent HZ compared with older adults [9]. A global systematic review reported significantly higher HZ risks in adults with chronic conditions, with pooled risk ratios (RRs) and 95% confidence intervals (CIs), for COPD, 1.41 (1.28–1.55); chronic kidney disease (CKD), 1.29 (1.10–1.51); asthma, 1.24 (1.16–1.31); diabetes mellitus (DM), 1.24 (1.14–1.35); depression, 1.23 (1.11–1.36); physical trauma, 2.01 (1.39–2.91); and psychological stress, 1.47 (1.03–2.10) [4]. Furthermore, a study in adults ≥50 YoA in Italy found that severe HZ requiring hospitalization may worsen chronic conditions [11]. Over 50% of US adults, an estimated 129 million people, had ≥1 major chronic condition in 2018 [12, 13]. Among these, 42% and 12% had ≥2 and ≥5 chronic conditions, respectively, with substantial costs to the healthcare system [13].

Most HZ studies in the US have focused on adults ≥50 YoA. Recent evidence is lacking on HZ incidence in adults with comorbidities, and criteria used to define these populations vary. The aim of this study was to estimate HZ incidence rates (IRs) in adults aged 18–49 YoA with comorbid conditions, ie, DM, COPD, asthma, depression, CKD, trauma, and stress (and without specific immunocompromising conditions [ICs] or autoimmune diseases [AIDs]). The HZ IR in each age and comorbid disease group was compared to the HZ IR in the 50–59 YoA immunocompetent population without comorbid conditions (ie, a group within the ≥50 YoA immunocompetent population for which RZV vaccination is currently recommended). This comparator group was selected to represent the youngest immunocompetent group currently eligible for vaccination, in order to determine how the risk of HZ in younger adults with comorbidities compares with the risk in this group. As younger age groups with ICs/AIDs are already eligible for vaccination, the study focused on comparisons with immunocompetent individuals. The proportions of adults with PHN and HZ hospitalization in each age and comorbidity group and the immunocompetent group were also estimated.

## METHODS

### Study Design and Populations

A retrospective cohort study was conducted using Merative MarketScan Commercial/Medicare Supplemental insurance databases (from 1 October 2015 to 31 March 2022), to estimate age-specific HZ IRs per 1000 person-years (PY) in adults with comorbid conditions. The Merative databases comprise a nationally representative sample of the US population covered by employer-sponsored health insurance [14], with deidentified, individual patient-level data and claims across various medical services (eg, inpatient, outpatient, pharmacy, enrollment, and laboratory tests) across all care settings.

Of the 64 811 481 participants in the Merative databases at the time of analyses, the overall study population was defined as adults ≥18 YoA, with no history of HZ or HZ vaccination, and with 15-month continuous enrollment (considering a grace period of 45 days) in the database (baseline period). The index date was day 1 of the 16th month of continuous enrollment. Participants were followed until the first occurrence of HZ or a censoring event: HZ vaccination, loss of coverage, or end of study (31 March 2022).

From the overall study population, adults were first excluded with a specific set of ICs/AIDs. The specific ICs excluded were human immunodeficiency virus, hematological malignancy, stem cell transplant, solid organ transplant, and solid tumors. The specific AIDs excluded were inflammatory bowel disease, multiple sclerosis, psoriatic arthritis, psoriasis, rheumatoid arthritis, and systemic lupus erythematosus. Next, the following comorbidity study populations were included for analysis: asthma, CKD, COPD, DM, depression, stress, and trauma. These comorbid conditions were chosen because they are at elevated risk for HZ and all, except stress and trauma, and had validated claims data algorithms with high positive predictive values [15–20]. As stress and trauma did not have well-validated claims data algorithms, these were included as exploratory analyses. In addition to the censoring events defined for the overall population, follow-up for individuals in the comorbidity population was censored if they developed a specific IC or AID (ie, at the first occurrence of any specified IC/AID).

**Figure 1.**
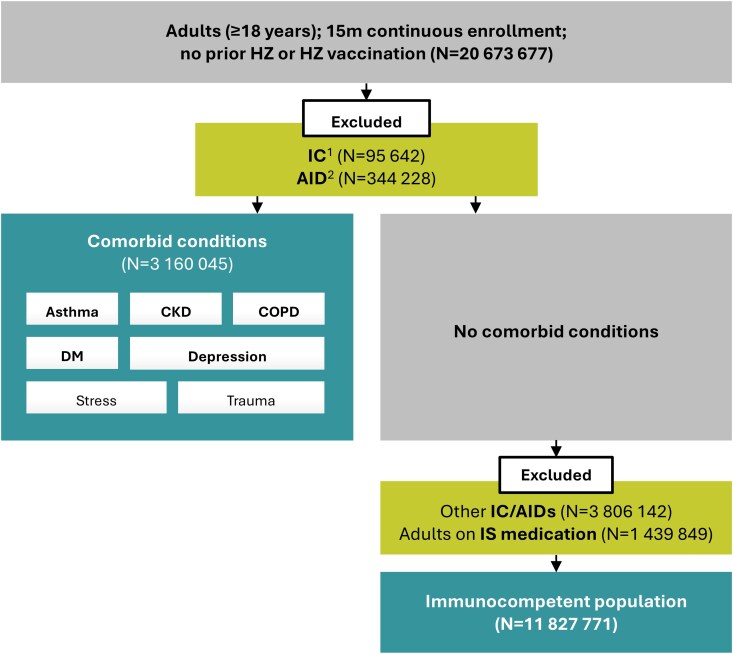
Derivation of comorbid disease and immunocompetent populations. Abbreviations: AID, autoimmune disease; CKD, chronic kidney disease; COPD, chronic obstructive pulmonary disease; DM, diabetes mellitus; HZ, herpes zoster; IC, immunocompromising condition; IS, immunosuppressive; m, month; N, number in group. Note: As stress and trauma populations were difficult to define, these were included as exploratory analyses. ^a^IC: solid tumors, hematological malignancies, solid organ transplant, human immunodeficiency virus, stem cell transplant; ^b^AIDs: multiple sclerosis, systemic lupus erythematosus, rheumatoid arthritis, inflammatory bowel disease, psoriasis, psoriatic arthritis.

To define the comparator population of immunocompetent adults, we selected the population without comorbid conditions (and without ICs/AIDs) and then excluded individuals with any other IC/AID or who were on immunosuppressive medication. In addition to the censoring events for the overall population, the immunocompetent population was censored at the first occurrence of any specified IC/AID condition, comorbid condition, or other IC/AID or immunosuppressive medication (Figure 1).

Adults with comorbid conditions had ≥1 comorbid condition during the baseline period, defined using International Classification of Diseases 10th revision (ICD-10) codes, medical resource use, and prescriptions. Herpes zoster was defined using ICD-10 codes (B02.2x) from hospital, emergency department, or ambulatory visit diagnoses and from oral antiviral dispensing (acyclovir, valacyclovir, or famciclovir) within 7 days before or after HZ diagnosis. Postherpetic neuralgia was defined using ≥1 subsequent diagnosis code B02.2x (any position) in the 90–180 days after HZ and ≥1 of the following: (1) ≥1 incident dispensing for anti-PHN medications in the 0–60 days after the first HZ diagnosis, without an anti-PHN medication in this medication class in the 365 days prior to initial HZ; (2) an ICD-10 diagnosis B02.2x (HZ with other nervous system involvement) in the 90–180 days after the HZ event; or (3) a new ICD-10 diagnosis M79.2 (neuralgia and neuritis, unspecified) or M54.10 (radiculopathy, site unspecified) in the 0–180 days after HZ, without neuralgia or radiculopathy in the 365 days prior to HZ. Herpes zoster hospitalizations were defined as HZ diagnosed in the hospital setting.

Age was treated as a time-varying variable and recalculated annually, with participants assigned to subsequent age groups as necessary. Analyses were stratified by the following approximate 10-year age groups: 18–29, 30–39, 40–49, 50–59, 60–69, 70–79, and ≥80 YoA.

### Analysis

Baseline population characteristics were described overall and by age group. Categorical variables were presented as absolute numbers and percentages. Continuous variables were presented as mean with standard deviation and/or median with interquartile ranges.

Incident HZ cases were divided by total patient-years at risk. Person-time at risk was defined as total follow-up time, starting from the index date until the occurrence of the outcome of interest or other censoring events. The exact Poisson 95% CIs of the IRs were computed. The HZ IRs in comorbidity populations <50 YoA were compared with the HZ IR in the immunocompetent 50–59 YoA population without comorbid conditions (reference group).

Incidence rate ratios (IRRs) compared HZ IRs in each comorbidity population with the reference group (immunocompetent population 50–59 YoA). Incidence rate ratios were calculated by dividing the HZ IRs by the reference group HZ IR. Adjusted IRRs (aIRRs) and 95% CIs were estimated using Poisson models, adjusting for covariates (eg, age, sex, number of hospitalizations, region, health plan type, medication, and comorbidities).

Using a previously defined [21] predetermined noninferiority margin of 0.62, the HZ IR for each population age group was classified as significantly higher (if the aIRR 95% CI lower limit [LL] was >1.0), comparable (ie, noninferior, if the aIRR 95% CI LL was >0.62 and ≤1.0), or inconclusive (for any other result) compared with the reference group. The noninferiority margin of 0.62 was derived from an IRR of 6.7/10.8, which represents the HZ incidence rates in the 50–59-year-old (6.7) and 60–69-year-old (10.8) populations. At the time, vaccination was recommended by the Centers for Disease Control and Prevention (CDC) from 60 YoA, and the Food and Drug Administration (FDA) had approved vaccination for adults 50–59 YoA with live-attenuated HZ vaccine [21].

The proportion of HZ cases developing PHN was the number with a first PHN event divided by all HZ cases with ≥180 days of continuous enrollment following HZ. The proportion of hospitalized HZ cases was the number with a first HZ hospitalization (HZ date from hospital visit only) divided by all HZ cases (HZ date from hospital, emergency department, or ambulatory visit) diagnosed from the index date until the censoring event.

Analyses were conducted in SAS version 9.04.01.

### Sensitivity Analyses

Sensitivity analyses assessed HZ IRs for each comorbid condition in adults with only 1 comorbid condition (eg, only DM or only asthma), whereas the main analysis included adults with 1 or more comorbid conditions and HZ IRs by age group in adults with either 1, 2, or ≥3 comorbid conditions.

## RESULTS

### Population and Baseline Characteristics

The overall population included 20 673 677 adults ≥18 YoA, with no history of HZ or HZ vaccination, and with 15 months of continuous enrollment. After excluding around 0.5% of adults with IC and around 1.7% with an AID, there were 3 160 045 adults with comorbidities. Among adults without comorbidities, and excluding those with other ICs/AIDs or on immunosuppressive medication, the immunocompetent population comprised 11 827 771 adults (Figure 1).

Across the comorbid conditions, the mean age at baseline ranged from 39.9 years for stress (28.6% male) to 68.5 years for CKD (55.7% male) (Supplementary Table 1). The percentage with ≥1 hospitalization during the baseline period ranged from 8.8% for asthma to 32.9% for CKD. Roughly 1% of each population had coronavirus disease 2019 (COVID-19) (range: 0.7%–1.5%). Use of preventive cancer screening services ranged from 57.6% for DM to 66.1% for asthma. Hypertension was common in all comorbid populations, ranging from 25.4% for stress to 91.8% for CKD (data not shown). Overall, in the immunocompetent 50–59 YoA comparison population at baseline, 52.2% were male, few had been hospitalized (1.3%) or had COVID-19 (0.4%), 45.1% had used preventive cancer screening services (Supplementary Table 1), and the most common health condition was hypertension (21.6%) followed by hyperthyroidism (5.7%).

### Herpes Zoster Incidence Rates

**Figure 2.**
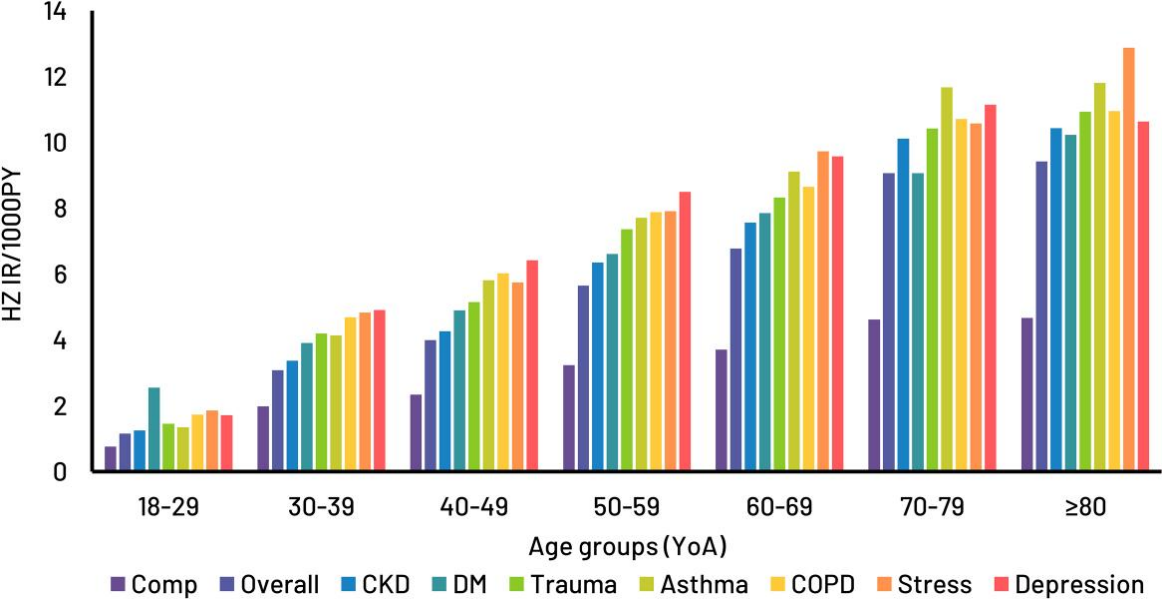
Herpes zoster incidence (per 1000 person-years [PY]) in overall, immunocompetent, and comorbid condition populations, by age group. Abbreviations: CKD, chronic kidney disease; Comp, immunocompetent; COPD, chronic obstructive pulmonary disease; DM, diabetes mellitus; HZ, herpes zoster; IR, incidence rate; PY, person-years; YoA, years of age.

Overall, 31 995 HZ events were reported in adults with comorbidities. Herpes zoster IRs increased with age and appeared higher in adults with comorbidities compared with immunocompetent and overall populations of the same age group (Figure 2; Supplementary Table 2). There were wide CIs for stress ≥70 YoA due to low numbers of HZ events (IR 10.6 [95% CI 7.0–15.3] in 70–79 YoA and IR 12.9 [95% CI 4.7–28.0] in ≥80 YoA) (Supplementary Table 2).

### Herpes Zoster Incidence Rate Ratios

**Figure 3.**
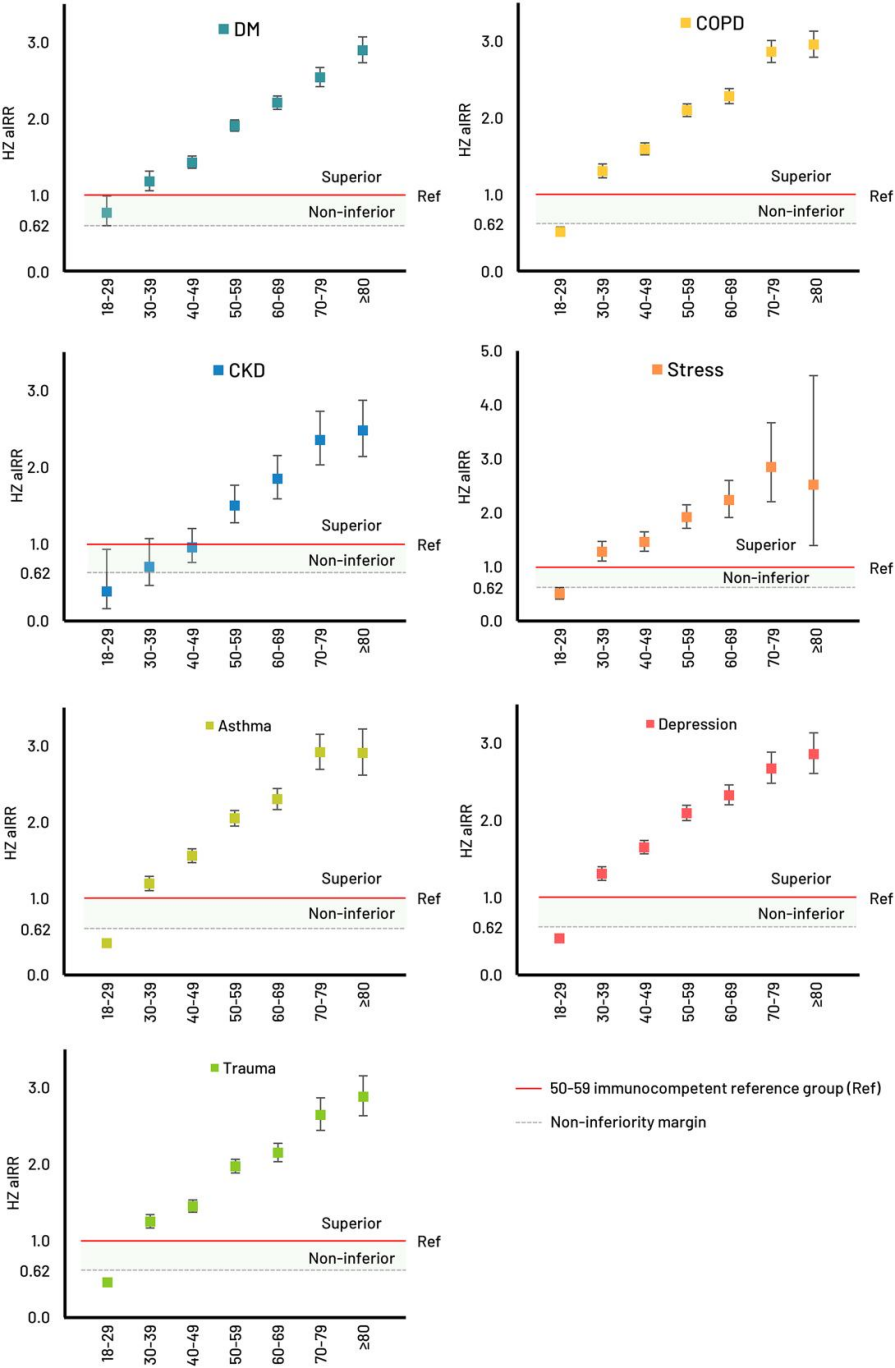
Herpes zoster (HZ) incidence rate ratios (IRRs) in comorbid condition populations by age group versus immunocompetent 50–59 years of age. Abbreviations: 95% CI, 95% confidence interval; aIRR, adjusted incidence rate ratio; CKD, chronic kidney disease; COPD, chronic obstructive pulmonary disease; DM, diabetes mellitus; HZ, herpes zoster; LL, lower limit; YoA, years of age. Note: The shaded area denotes findings comparable to the reference group (ie, 95% confidence interval lower limit [CI LL] > 0.62 and ≤1.0); above red line indicates findings significantly higher than reference group (ie, 95% CI LL > 1.0).

Compared with the reference group (immunocompetent 50–59 YoA), and using the predetermined noninferiority margin of 0.62 for the aIRR 95% CI LL, the 95% CI LLs indicated that HZ IRs were noninferior and categorized as significantly higher (95% CI LL >1.0) from 30 to 39 YoA and older in adults with asthma, COPD, depression, DM, stress, and trauma (30–39 YoA aIRR 95% CI LLs were 1.10, 1.22, 1.22, 1.06, 1.11, and 1.17, respectively). Herpes zoster IRs were also noninferior and categorized as comparable (95% CI LL > 0.62 and ≤1.0) in CKD 40–49 YoA and significantly higher in CKD 50–59 YoA and older (aIRR 95% CI LL of 0.76 and 1.28, respectively) (Figure 3; Supplementary Table 2).

### Postherpetic Neuralgia and Herpes Zoster Hospitalization

Among adults with comorbidities, 921 developed PHN, and 304 were hospitalized for HZ.

**Figure 4.**
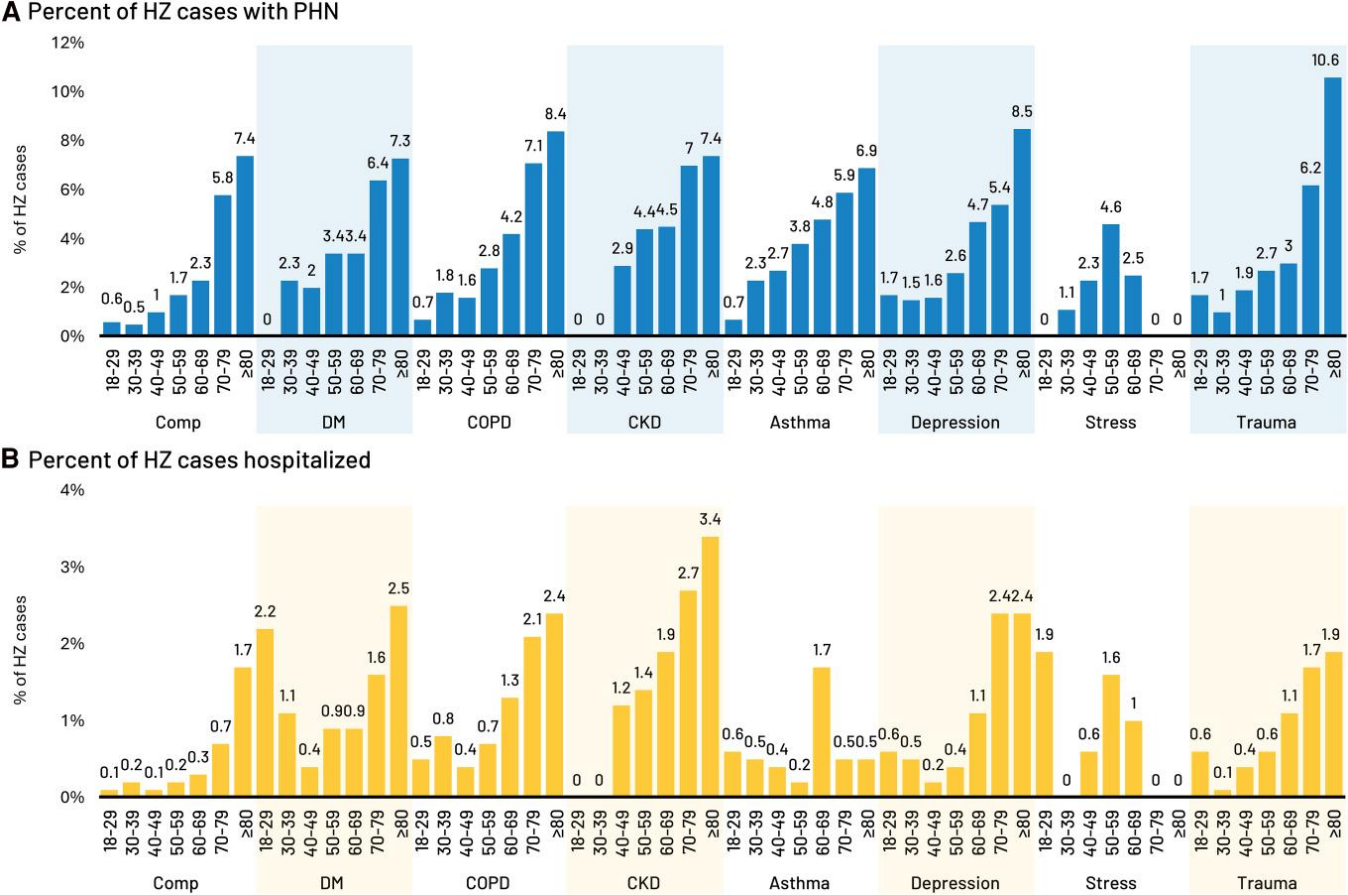
Percent of herpes zoster cases (*A*) with PHN and (*B*) with HZ hospitalization, by population and age group. Abbreviations: CKD, chronic kidney disease; Comp, immunocompetent; COPD, chronic obstructive pulmonary disease; DM, diabetes mellitus; HZ, herpes zoster; PHN, postherpetic neuralgia.

The proportions developing PHN generally appeared higher in older age groups, increasing with age from 40–49 YoA onwards, eg, from 1.6% to 8.4% in 40–49 YoA to ≥80 YoA with COPD (Figure 4). For most comorbidities, a higher proportion developed PHN compared with the same immunocompetent age groups.

The proportions of adults with HZ hospitalizations also generally appeared higher in older age groups and tended to increase with age from 60–69 YoA onwards, except in the DM population that had high HZ hospitalization in 18–29 YoA. Among adults ≥60 YoA, HZ hospitalization proportions varied between 0.3% and 1.7% in immunocompetent adults, while proportions were higher in adults with comorbidities, eg, 0.9%–2.5% for DM, 1.3%–2.4% for COPD, 1.9%–3.4% for CKD, 1.1%–2.4% for depression, and 1.1%–1.9% for trauma. For adults with asthma, the highest proportion with HZ hospitalizations was in 60–69 YoA (1.7%), and for stress, in 18–29 YoA (1.9%) (Figure 4). Some comorbidities had small numbers of HZ cases for some or all age groups, which affected calculation of proportions.

### Sensitivity Analyses

**Figure 5.**
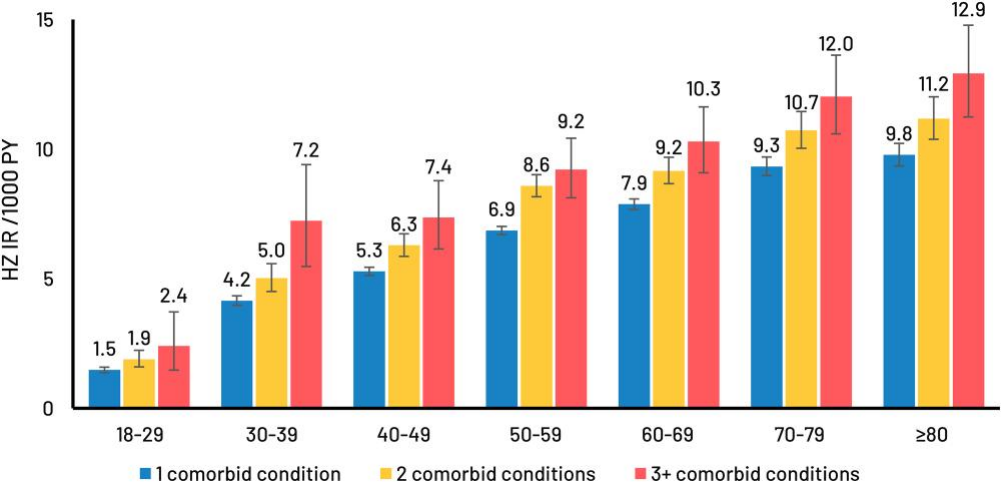
Herpes zoster incidence rate by number of comorbid conditions and age group. Abbreviations: HZ, herpes zoster; IR, incidence rate; PY, person-years; YoA, years of age.

In sensitivity analyses, HZ IRs and IRRs were investigated for each comorbid condition in subpopulations with only 1 comorbid condition at baseline. Applying the predefined noninferiority margin of 0.62, the aIRR 95% CI results indicated that HZ IRs were similar to the main analysis findings, except among 30–39 YoA with only DM or only asthma, which had comparable HZ IRs to the reference group (instead of significantly higher in the main analysis). In a sensitivity analysis by number of comorbid conditions, the HZ IR appeared to increase by age group and in adults with more comorbid conditions (Figure 5).

## DISCUSSION

This observational cohort study, conducted from 2015 to 2022, estimated the HZ burden by approximate 10-year age groups in US adults with comorbid conditions (and without selected ICs/AIDs). For asthma, COPD, depression, DM, stress, and trauma, HZ IRs were significantly higher for 30–39 YoA and older versus immunocompetent 50–59 YoA. For CKD, the HZ IR in 40–49 YoA was comparable to the 50–59 YoA immunocompetent population and was significantly higher from the age of 50, possibly because CKD typically becomes more prevalent in adults ≥40 YoA, and numbers were small in younger age groups [22]. As expected, the HZ IR increased with age in all populations and appeared higher in adults with comorbid conditions compared with immunocompetent people and the overall population (ie, total population before excluding ICs/AIDs or comorbidities) of the same age group. Adults with comorbid conditions also appeared to generally have higher rates of PHN and HZ hospitalizations compared with the immunocompetent population and overall population of the same age groups. In the stress population, the generalizability of results may be affected by the small numbers included in some subgroups, eg, for ≥70 YoA assessed for PHN or HZ hospitalization. Finally, HZ IRs appeared to increase in adults with multiple comorbidities in all age groups.

While it is possible that significantly higher HZ rates in adults with comorbidities may partly be due to more frequent contact with health services versus 50–59 YoA immunocompetent adults, our results were adjusted for potential confounders such as other baseline conditions that may also drive more frequent contact with health services (eg, other comorbidities such as hypertension or renal disease). Additionally, our findings are in line with other studies showing a higher risk of HZ in groups with comorbidities. While other studies typically presented results for all ages [4, 23, 24], or <50 YoA combined [9, 25], they all compared comorbid disease populations to noncomorbid disease populations within the same age group. A global systematic review and meta-analysis confirmed a significantly higher HZ risk in adults (all ages) with COPD, CKD, asthma, DM, depression, stress, and trauma [4]. Similarly, a cohort study in Spain (2009–2012) identified a significantly higher risk of HZ (all ages) in adults with COPD, asthma, and DM [23]. In the US, a case–control study (commercial claims from 2007) reported significantly higher HZ risks in adults (20–64 YoA) with COPD, DM, and depression. Among adults <50 YoA, a cohort study in Germany (2018) found significantly higher HZ risks in adults with asthma and depression [9]; while a United Kingdom case–control study (2000–2011) reported a similar finding for adults with CKD, asthma, DM, and depression [25]. One study in the Republic of Korea reported HZ risks in adults by 10-year age groups [10] and found that among adults 18–49 YoA, HZ incidence was 29%–37% higher for COPD and 25%–39% higher for DM, while it was 32%–36% higher for adults 30–49 YoA with asthma. In this study, the CKD data showed a trend toward a higher HZ risk (IRRs between 1.23 and 1.55) in age groups <50 YoA [10].

Our study addressed evidence gaps on HZ risk in adults 18–49 YoA with comorbidities. Each comorbidity was carefully selected after rigorously excluding adults with a range of specific ICs and AIDs. Therefore, the HZ IRs may be lower than those assessed in the entire population with comorbid conditions. While most algorithms used to identify participants with ICs/AIDs and comorbid conditions had demonstrated a good positive predictive value, misclassification was expected for trauma and stress algorithms. Very low numbers in some subgroups with stress make it difficult to generalize those results. To improve confidence in the definitions, similar algorithms were used in other studies of comorbid, IC/AID populations.

Key strengths included the large study size, and the findings are generalizable to US adults with commercial/Medicare Supplemental health insurance. As with other claims database studies, limitations relate to generalizability of findings to noncommercial/Medicare Supplemental insured populations and possible underreporting of certain events and diagnoses, with no direct data on deaths. There may have been unmeasured confounding factors, such as disease severity and duration, which could be linked to an increased risk of HZ. However, these factors are difficult to measure using administrative claims databases and may not be captured. Our study assessed proxies for disease activity, such as medication use and healthcare utilization for disease severity, to measure confounding.

Our study demonstrated the higher incidence of HZ and its complications in adults with comorbidities. The results underscore the importance of continuing to recommend HZ vaccination for adults ≥50 YoA, particularly those with underlying comorbidities. As these populations are likely to receive care from diverse specialists, the findings could guide primary care physicians in identifying patients at higher risk of HZ who could benefit from vaccination. In addition, the results could support discussions on the possible need to vaccinate younger age groups with comorbid conditions.

## CONCLUSIONS

Currently, RZV vaccination is recommended for all adults ≥50 YoA in the US, regardless of comorbid conditions, and for immunocompromised adults ≥19 YoA. Our findings indicate that younger immunocompetent adults, beginning at age 30, with certain comorbidities have a high HZ incidence. The HZ IR in younger adults was significantly higher than in immunocompetent individuals 50–59 YoA without comorbidities, one of the groups within the population for which RZV vaccination is currently recommended. Our results highlight the importance of vaccinating adults ≥50 YoA with comorbid conditions and could help inform discussions on vaccination needs in younger comorbid populations.

## Supplementary Material

ciag095_Supplementary_Data

## Supplementary Data

Supplementary materials are available at *Clinical Infectious Diseases* online. Consisting of data provided by the authors to benefit the reader, the posted materials are not copyedited and are the sole responsibility of the authors, so questions or comments should be addressed to the corresponding author.
