# Supplementary material for: Age-Specific Risk of Herpes Zoster in Adults Aged ≥18 Years With Comorbid Conditions—A Retrospective Cohort Study in the United States
Source: Clin Infect Dis. 2026 Mar 27;82(6):e1375–83. doi: 10.1093/cid/ciag095 (PMC13341258; doi:10.1093/cid/ciag095)
Supplement: ciag095_Supplementary_Data [file ciag095_supplementary_data.docx]

# Supplemental material

**Table S1.** Baseline characteristics – Comorbidity populations (all age groups) and comparison population Immunocompetent 50–59 YoA

**Table S2.** Herpes zoster incidence rates (95% CI) and adjusted incidence rate ratios (95% CI) by age group and population

**Table S1 Baseline characteristics – Comorbidity populations (all age groups) and comparison population Immunocompetent 50–59 YoA**

|  | **Comorbidity populations, all ages (18+ YoA)** | | | | | | | **Comparison population** |
| --- | --- | --- | --- | --- | --- | --- | --- | --- |
| a | **Asthma** | **CKD** | **COPD** | **Depression** | **DM** | **Stress** | **Trauma** | **Comp 50–59 YoA** |
|  |  |  |  |  |  |  |  |  |
| **N, index date** | 414 541 | 171 497 | 869 220 | 639 604 | 953 205 | 84 942 | 708 115 | 2 029 277 |
| **Mean age (SD)** | 44.4 (16.6) | 68.5 (15.3) | 54.8 (17.6) | 45.5 (17.2) | 58.9 (13.3) | 39.9 (14.0) | 45.3 (17.2) | 54.4 (2.8) |
| **Sex, n (%)** | | | | | | | | |
| Female | 263 908 (63.7) | 75 979 (44.3) | 516 428 (59.4) | 449 683 (70.3) | 440 286 (46.2) | 60 683 (71.4) | 362 170 (51.1) | 969 658 (47.8) |
| Male | 150 633 (36.3) | 95 518 (55.7) | 352 792 (40.6) | 189 921 (29.7) | 512 919 (53.8) | 24 259 (28.6) | 345 945 (48.9) | 1 059 619 (52.2) |
| **Number of hospitalizations, n (%)** | | | | | | | | |
| 0 | 378 094 (91.2) | 115 104 (67.1) | 736 964 (84.8) | 522 829 (81.7) | 787 409 (82.6) | 70 108 (82.5) | 629 934 (89.0) | 2 003 712 (98.7) |
| 1 | 28 455 (6.9) | 35 059 (20.4) | 94 397 (10.9) | 82 116 (12.8) | 121 406 (12.7) | 9869 (11.6) | 55 038 (7.8) | 23 376 (1.2) |
| 2 | 5344 (1.3) | 13 040 (7.6) | 24 933 (2.9) | 21 188 (3.3) | 29 391 (3.1) | 2755 (3.2) | 14 359 (2.0) | 1909 (0.1) |
| ≥3 | 2648 (0.6) | 8294 (4.8) | 12 926 (1.5) | 13 471 (2.1) | 14 999 (1.6) | 2210 (2.6) | 8784 (1.2) | 280 (0.0) |
| **Health plan type, n (%)** | | | | | | | | |
| Commercial | 375 182 (90.5) | 74 385 (43.4) | 650 270 (74.8) | 565 526 (88.4) | 705 162 (74.0) | 81 437 (95.9) | 638 498 (90.2) | 2 027 910 (99.9) |
| Medicare | 39 359 (9.5) | 97 112 (56.6) | 218 950 (25.2) | 74 078 (11.6) | 248 043 (26.0) | 3505 (4.1) | 69 617 (9.8) | 1367 (0.1) |
| **Geographical region, n (%)** | | | | | | | | |
| North central | 94 754 (22.9) | 53 460 (31.2) | 216 947 (25.0) | 160 080 (25.0) | 235 686 (24.7) | 18 384 (21.6) | 153 714 (21.7) | 397 167 (19.6) |
| South | 165 688 (40.0) | 73 105 (42.6) | 392 645 (45.2) | 286 214 (44.7) | 467 488 (49.0) | 33 670 (39.6) | 281 296 (39.7) | 914 470 (45.1) |
| West | 66 500 (16.0) | 18 946 (11.0) | 97 747 (11.2) | 93 923 (14.7) | 109 308 (11.5) | 17 284 (20.3) | 118 813 (16.8) | 359 499 (17.7) |
| Northeast | 86 768 (20.9) | 25 586 (14.9) | 160 540 (18.5) | 98 173 (15.3) | 138 315 (14.5) | 15 384 (18.1) | 152 598 (21.5) | 349 928 (17.2) |
| Missing/Unknown | 831 (0.2) | 400 (0.2) | 1341 (0.2) | 1214 (0.2) | 2408 (0.3) | 220 (0.3) | 1694 (0.2) | 8213 (0.4) |
| **Preventive services use, n (%)** | 274 159 (66.1) | 99 326 (57.9) | 556 011 (64.0) | 417 944 (65.3) | 549 444 (57.6) | 53 273 (62.7) | 421 651 (59.5) | 91 4461 (45.1) |
| **COVID-19, n (%)** | 5225 (1.3) | 1604 (0.9) | 7856 (0.9) | 7124 (1.1) | 8983 (0.9) | 1295 (1.5) | 5220 (0.7) | 8158 (0.4) |
| **Comorbidities affecting >10%, n (%)** | | | | | | | | |
| Hypertension | 135 789 (32.8) | 157 474 (91.8) | 438 187 (50.4) | 229 526 (35.9) | 743 561 (78.0) | 21 584 (25.4) | 221 348 (31.3) | 438 552 (21.6) |
| Hyperthyroidism | 47 252 (11.4) | 36 334 (21.2) | 136 978 (15.8) | 92 866 (14.5) | 155 193 (16.3) | 9 702 (11.4) | 75 014 (10.6) |  |
| CVD |  | 30 816 (18.0) |  |  |  |  |  |  |
| CLD | 414 541 (100) | 32 886 (19.2) | 526 263 (60.5) | 81 238 (12.7) | 110 069 (11.5) | 10 785 (12.7) | 73 264 (10.3) |  |
| CHF |  | 44 685 (26.1) |  |  |  |  |  |  |
| PVD |  | 19 944 (11.6) |  |  |  |  |  |  |
| Renal disease |  | 171 497 (100) | 123 122 (14.2) |  | 191 602 (20.1) |  |  |  |
| **Index date by year, n (%)** | | | | | | | | |
| 2017 | 232 558 (56.1) | 118 844 (69.3) | 56 7071 (65.2) | 356 749 (55.8) | 617 720 (64.8) | 39 415 (46.4) | 451 971 (63.8) | 1 259 648 (62.1) |
| 2018 | 52 908 (12.8) | 11 499 (6.7) | 86 018 (9.9) | 76 781 (12.0) | 94 287 (9.9) | 10 433 (12.3) | 83 138 (11.7) | 237 343 (11.7) |
| 2019 | 56 186 (13.6) | 18 537 (10.8) | 101 423 (11.7) | 85 605 (13.4) | 112 614 (11.8) | 12 244 (14.4) | 83 318 (11.8) | 250 714 (12.4) |
| 2020 | 33 685 (8.1) | 6180 (3.6) | 46 927 (5.4) | 52 685 (8.2) | 51 428 (5.4) | 10 079 (11.9) | 42 547 (6.0) | 136 822 (6.7) |
| 2021 | 35 470 (8.6) | 15 899 (9.3) | 62 990 (7.2) | 61 042 (9.5) | 71 974 (7.6) | 11 249 (13.2) | 42 771 (6.0) | 132 590 (6.5) |
| 2022 | 3734 (0.9) | 538 (0.3) | 4791 (0.6) | 6742 (1.1) | 5182 (0.5) | 1522 (1.8) | 4370 (0.6) | 12 160 (0.6) |

Abbreviations: CHF, congestive heart failure; CKD, chronic kidney disease; CLD, chronic lung disease; Comp, immunocompetent; COPD, chronic obstructive pulmonary disease; COVID-19, coronavirus disease 2019; CVD, cerebrovascular disease; DM, diabetes mellitus; n/N, number in group; PVD, peripheral vascular disease; SD, standard deviation; YoA, years of age

**Table S2 Herpes zoster incidence rate (95% CI) and adjusted incidence rate ratios (95% CI) by age group and population**

| **Population** | | **Age group (YoA)** | | | | | | |
| --- | --- | --- | --- | --- | --- | --- | --- | --- |
|  |  | **18–29** | **30–39** | **40–49** | **50–59** | **60–69** | **70–79** | **≥80** |
| Overall | n | 8353 | 20 269 | 30 848 | 45 680 | 28 195 | 10 065 | 6974 |
|  | N | 4 988 946 | 4 489 379 | 4 661 776 | 4 792 663 | 2 835 938 | 743 756 | 494 944 |
|  | PY | 7 189 663 | 6 572 941 | 7 722 698 | 8 092 133 | 4 161 466 | 1 110 738 | 740 630 |
|  | IR [95% CI] | 1.16 [1.14–1.19] | 3.08 [3.04–3.13] | 3.99 [3.95–4.04] | 5.65 [5.59–5.70] | 6.78 [6.70–6.86] | 9.06 [8.89–9.24] | 9.42 [9.20–9.64] |
| Comp | n | 3342 | 6805 | 7719 | 9251 | 4564 | 855 | 479 |
|  | N | 3 629 744 | 2 902 845 | 2 593 703 | 2 259 519 | 1 082 484 | 174 433 | 99 648 |
|  | PY | 4 369 363 | 3 418 681 | 3 288 415 | 2 858 838 | 1 229 766 | 185 165 | 102 630 |
|  | IR [95% CI] | 0.77 [0.74–0.79] | 1.99 [1.94–2.04] | 2.35 [2.30–2.40] | 3.24 [3.17–3.30] | 3.71 [3.60–3.82] | 4.62 [4.31–4.94] | 4.67 [4.26–5.11] |
| CKD | n | <11 | 21 | 82 | 282 | 411 | 548 | 708 |
|  | N | 1960 | 4925 | 13 555 | 30 408 | 42 187 | 40 707 | 50 630 |
|  | PY | 2385 | 6231 | 19 227 | 44 374 | 54 355 | 54 167 | 67 816 |
|  | IR [95% CI] | 1.26 [0.26–3.68] | 3.37 [2.09–5.15] | 4.27 [3.39–5.29] | 6.36 [5.64–7.14] | 7.56 [6.85–8.33] | 10.12 [9.29–11.00] | 10.44 [9.69–11.24] |
|  | aIRR [95% CI] | 0.39 [0.16–0.93] | 0.70 [0.46–1.07] | 0.96 [0.76–1.20] | 1.50 [1.28–1.77] | 1.85 [1.59–2.15] | 2.36 [2.03–2.73] | 2.48 [2.14–2.87] |
| DM | n | 45 | 266 | 1159 | 3264 | 3108 | 1681 | 1140 |
|  | N | 14 472 | 51 636 | 159 902 | 320 996 | 291 504 | 133 101 | 80 926 |
|  | PY | 17 636 | 67 947 | 236 522 | 493 490 | 395 901 | 185 445 | 111 392 |
|  | IR [95% CI] | 2.55 [1.86–3.41] | 3.92 [3.46–4.42] | 4.90 [4.62–5.19] | 6.61 [6.39–6.85] | 7.85 [7.58–8.13] | 9.07 [8.64–9.51] | 10.23 [9.65–10.85] |
|  | aIRR [95% CI] | 0.78 [0.61–0.99] | 1.18 [1.06–1.32] | 1.43 [1.35–1.52] | 1.91 [1.84–1.99] | 2.21 [2.13–2.30] | 2.54 [2.42–2.67] | 2.90 [2.73–3.07] |
| Trauma | n | 356 | 740 | 1276 | 2143 | 1244 | 474 | 421 |
|  | N | 160 133 | 122 890 | 153 895 | 178 295 | 106 086 | 31 530 | 27 828 |
|  | PY | 242 797 | 176 201 | 247 311 | 290 932 | 149 547 | 45 450 | 38 491 |
|  | IR [95% CI] | 1.47 [1.32–1.63] | 4.20 [3.90–4.51] | 5.16 [4.88–5.45] | 7.37 [7.06–7.69] | 8.32 [7.86–8.79] | 10.43 [9.51–11.41] | 10.94 [9.92–12.03] |
|  | aIRR [95% CI] | 0.46 [0.42–0.51] | 1.25 [1.17–1.34] | 1.45 [1.38–1.53] | 1.97 [1.89–2.06] | 2.15 [2.03–2.27] | 2.65 [2.44–2.87] | 2.88 [2.63–3.15] |
| Asthma | n | 180 | 428 | 810 | 1127 | 714 | 365 | 194 |
|  | N | 96 312 | 78 023 | 92 591 | 96 333 | 58 816 | 21 636 | 11 304 |
|  | PY | 132 901 | 103 436 | 139 432 | 146 333 | 78 393 | 31 265 | 16 425 |
|  | IR [95% CI] | 1.35 [1.16–1.57] | 4.14 [3.76–4.55] | 5.81 [5.42–6.22] | 7.70 [7.26–8.17] | 9.11 [8.45–9.80] | 11.68 [10.51–12.94] | 11.81 [10.21–13.60] |
|  | aIRR [95% CI] | 0.42 [0.37–0.47] | 1.19 [1.10–1.29] | 1.56 [1.47–1.65] | 2.05 [1.95–2.15] | 2.30 [2.17–2.44] | 2.91 [2.69–3.15] | 2.90 [2.62–3.22] |
| COPD | n | 205 | 617 | 1439 | 2746 | 2279 | 1595 | 1250 |
|  | N | 84 695 | 95 615 | 155 200 | 227 621 | 197 403 | 108 837 | 84 428 |
|  | PY | 118 174 | 131 680 | 239 000 | 348 505 | 263 550 | 148 865 | 114 084 |
|  | IR [95% CI] | 1.74 [1.51–1.99] | 4.69 [4.32–5.07] | 6.02 [5.71–6.34] | 7.88 [7.59–8.18] | 8.65 [8.30–9.01] | 10.71 [10.20–11.25] | 10.96 [10.36–11.58] |
|  | aIRR [95% CI] | 0.52 [0.46–0.58] | 1.31 [1.22–1.40] | 1.60 [1.52–1.68] | 2.10 [2.02–2.18] | 2.28 [2.19–2.38] | 2.86 [2.72–3.01] | 2.96 [2.79–3.13] |
| Stress | n | 53 | 125 | 164 | 192 | 96 | 28 | <11 |
|  | N | 23 902 | 21 787 | 20 639 | 16 611 | 7413 | 1877 | 352 |
|  | PY | 28 432 | 25 863 | 28 518 | 24 249 | 9871 | 2645 | 466 |
|  | IR [95% CI] | 1.86 [1.40–2.44] | 4.83 [4.02–5.76] | 5.75 [4.90–6.70] | 7.92 [6.84–9.12] | 9.73 [7.88–11.88] | 10.59 [7.04–15.30] | 12.88 [4.73–28.03] |
|  | aIRR [95% CI] | 0.50 [0.41–0.62] | 1.28 [1.11–1.47] | 1.46 [1.29–1.65] | 1.92 [1.71–2.15] | 2.23 [1.92–2.60] | 2.85 [2.21–3.67] | 2.52 [1.39–4.54] |
| Depression | n | 315 | 755 | 1323 | 1816 | 1139 | 540 | 379 |
|  | N | 139 207 | 121 796 | 140 359 | 144 710 | 90 695 | 35 653 | 26 878 |
|  | PY | 182 845 | 153 755 | 205 975 | 213 802 | 119 071 | 48 462 | 35 618 |
|  | IR [95% CI] | 1.72 [1.54–1.92] | 4.91 [4.57–5.27] | 6.42 [6.08–6.78] | 8.49 [8.11–8.89] | 9.57 [9.02–10.14] | 11.14 [10.22–12.12] | 10.64 [9.60–11.77] |
|  | aIRR [95% CI] | 0.48 [0.43–0.53] | 1.31 [1.22–1.40] | 1.65 [1.57–1.74] | 2.09 [2.00–2.19] | 2.33 [2.20–2.46] | 2.67 [2.48–2.88] | 2.86 [2.61–3.13] |
| 1 comorbidity | IR [95% CI] | 1.5 [1.4–1.6] | 4.2 [4.0–4.3] | 5.3 [5.1–5.4] | 6.9 [6.7–7.0] | 7.9 [7.7–8.1] | 9.3 [9.0–9.7] | 9.8 [9.4–10.2] |
| 2 comorbidities | IR [95% CI] | 1.9 [1.6–2.2] | 5.0 [4.5–5.6] | 6.3 [5.9–6.7] | 8.6 [8.2–9.0] | 9.2 [8.7–9.7] | 10.7 [10.0–11.5] | 11.2 [10.4–12.0] |
| ≥3 comorbidities | IR [95% CI] | 2.4 [1.5–3.7] | 7.2 [5.5–9.4] | 7.4 [6.1–8.8] | 9.2 [8.1–10.4] | 10.3 [9.1–11.6] | 12.0 [10.6–13.6] | 12.9 [11.2–14.8] |

Abbreviations: 95% CI, 95% confidence interval; aIRR, adjusted incidence rate ratio; CKD, chronic kidney disease; Comp, immunocompetent; COPD, chronic obstructive pulmonary disease; COVID-19, coronavirus disease 2019; DM, diabetes mellitus; IR, incidence rate; n/N, number with HZ/total number in age group; PY, person-years; YoA, years of age

Notes aIRR: Adjusted for the following covariates in all models: age in categories, chronic pulmonary disease, geographic region, hyperthyroidism, index year, renal disease, sex, use of preventive services; and for the following covariates for each population:

- *CKD* - dementia, number of hospitalizations, osteoporosis;
- *DM* - chronic lung disease, dementia, any malignancy, myocardial infarction, moderate or severe liver disease;
- *Trauma* - cerebrovascular disease, chronic lung disease, COVID-19 diagnosis, dementia, hypertension, myocardial infarction, mild liver disease, metastatic solid tumor, osteoporosis, peripheral vascular disease;
- *Asthma* - moderate or severe liver disease, metastatic solid tumor, number of hospitalizations, osteoporosis, use of preventive services, rheumatic disease;
- *COPD* - dementia, hypertension, any malignancy, myocardial infarction, moderate or severe liver disease, osteoporosis, rheumatic disease;
- *Stress* - chronic lung disease, COVID-19 diagnosis, dementia, any malignancy, moderate or severe liver disease, osteoporosis; and
- *Depression* - cerebrovascular disease, chronic lung disease, COVID-19 diagnosis, dementia, myocardial infarction, moderate or severe liver disease, rheumatic disease.
